# Experimental and Mathematical Modelling Investigation of Plasma Electrolytic Oxidation (PEO) for Surface Hardening of 20Ch Steel

**DOI:** 10.3390/ma17246043

**Published:** 2024-12-10

**Authors:** Kuat Kombayev, Fuad Khoshnaw, Gulzhaz Uazyrkhanova, Gulzhaz Moldabayeva

**Affiliations:** 1 International School of Engineering, East Kazakhstan State Technical University, Ust-Kamenogorsk 070004, Kazakhstan; kkombaev@edu.ektu.kz (K.K.); guazyrhanova@edu.ektu.kz (G.U.); gmoldabaeva@edu.ektu.kz (G.M.); 2 School of Engineering and Sustainable Development, De Montfort University, Leicester LE1 9BH, UK

**Keywords:** plasma, hardening, modelling, microstructure, steel

## Abstract

This study aimed to develop an alternative surface hardening technique for low-carbon steel alloy type 20Ch using plasma electrolytic oxidation (PEO). The surface hardening of 20Ch alloy steel samples was achieved through PEO in a Na_2_CO_3_ electrolyte solution. Optimal processing parameters were determined experimentally by measuring voltage and applied current. Quenching was performed in the electrolyte stream, and plasma was ionised through excitation. A mathematical model based on thermal conductivity equations and regression analysis was developed to relate the key parameters of the hardening process. The results from both the experimental and mathematical models demonstrated that PEO significantly reduces hardening time compared to traditional methods. The microstructural images revealed the transformation of the coarse-grained pearlite–ferrite structure into quenched martensite. Vickers microhardness tests indicated a substantial increase in surface hardness after PEO treatment, compared to the untreated samples. The major advantages of PEO include lower energy consumption, high quenching rates, and the ability to perform localised surface treatments. These benefits contribute to overall cost reduction, making PEO a promising surface hardening method for various industrial applications.

## 1. Introduction

Enhancing surface hardness and improving wear resistance is crucial for engineering applications such as mining and oil and gas production. These improvements ensure the durability and efficiency of equipment operating in harsh environments [1]. One key operation of machine-building enterprises in the oil and gas sector is the production of pipeline valves and oil-field equipment. These valves are typically manufactured from low-carbon steel (LCS) alloys through machining, with components often welded together. For example, wedge column equipment strapping (CES), which plays a critical role in the oil pumping process, is subjected to high contact stresses and impact fatigue wear. To withstand these harsh conditions, the piping of wedge string equipment must be reinforced. Strengthening a CES die made from LCS type 20Ch, equivalent to American Iron and Steel Institute (AISI) 5120, as specified by GOST 33260-2015 [2], is commonly achieved using a traditional carburising process. This involves placing the die in an electric furnace and treating it with solid carburising, typically using charcoal, followed by quenching to enhance its hardness and durability [3].

Carburising and nitriding are diffusion-based processes in which controlled exposure over time is essential for enhancing the surface’s hardness and corrosion resistance. Researchers have used carbon, nitrogen, and other elements in solid, liquid, and gas media to achieve similar outcomes, increasing oxidation [4], hardness, and resistance to corrosion fatigue [5]. The carbon or nitrogen penetration rate during carburising or nitriding is relatively slow, requiring the workpiece to be heated in the carburising medium for a few hours at a specific temperature to achieve a case depth of 1–2 mm, followed by quenching [6]. However, these processes are associated with significant heat losses, which reduce overall efficiency. Additionally, they involve considerable time expenditure, particularly when heating the furnace to the required temperature, and are highly energy-intensive, with power consumption ranging from 60 to 100 kW [7]. In the past, CES dies made from LCS alloys were typically strengthened using this method, in which the surface is saturated with carbon through carburization, followed by quenching [8]. Although effective, this process is highly inefficient, demanding significant time, labour, and energy.

Kombayev et al. [9] presented a modified electro-plasma processing technology for strengthening the surface layer of LCS, which was proposed as an alternative to carburising and subsequent hardening. The process enhances the surface layer to a depth of 1000–1700 μm and increases microhardness by 1.5 to 2 times. The treatment results in a martensitic microstructure under a dark, modified surface layer, transitioning from the initial pearlite–ferrite structure. During this process, an interaction occurs between the steel 12Ch18N10T anode and the electrolyte, leading to the diffusion of elements such as chromium, nickel, and sodium from the anode and electrolyte to the surface of the cathode sample. Mass transfer processes are activated at high plasma temperatures, allowing atoms or ions from the electrolyte to migrate to the steel surface through thermal activity and ionisation. The method offers advantages such as low energy consumption, high hardening speed, localised surface treatment for complex shapes, improved wear resistance, and a smoother surface. Sagdoldina et al. [10] investigated the effects of electrolytic-plasma thermocycler hardening on the surface and microstructure of 40Ch medium-carbon steel, commonly used for tools and machine parts. The study utilised optical, scanning electron, X-ray diffraction, and microhardness measurements. The results showed that the treatment transformed the pearlite–ferrite structure into hardened martensite, with an optimal treatment time of 2 s yielding a hardening depth of 1.6 mm and a hardness increase to 966 HV. This process enhanced the steel’s surface hardening potential, improving its service life and reliability. Magazov et al. [11] studied the effects of ultrasonic nano surface treatment and electrolytic-plasma thermo-cyclic surface treatment on the wear resistance of AISI 52100 bearing steel. Friction tests conducted under dry conditions revealed that both treatments improved wear resistance, with UNSM at 700 °C and five cycles of EPSM showing the best results. Microstructural analysis indicated that EPSM produced residual austenite, while UNSM induced severe plastic deformation. The treatments also increased surface hardness, and a correlation was observed between wear volume, friction coefficient, and hardness.

Besides increasing hardness, PEO has been used to enhance the corrosion resistance of different metallic alloys. Talal A. Aljohani et al. [12] found that adding SiC to PEO coatings on AA2014 aluminium reduced corrosion rates by 99.8%, due to its compact structure. In magnesium alloys [13], adjusting PEO parameters improved coating density, increasing corrosion resistance eightfold compared to uncoated samples. These results underscore PEO’s potential to significantly improve alloy durability in corrosive settings.

Mathematical modelling enhances experimental approaches by predicting process behaviours, allowing researchers to refine parameters and reduce time, cost, and material waste before physical trials. Accordingly, combined experimental and modelling efforts have advanced plasma electrolytic oxidation (PEO) and heat treatment processes. While some researchers have experimentally analysed the effects of various electrolytes on PEO coatings, others have used computational models to optimise treatment processes. Together, these approaches improve industrial applications by enhancing efficiency and ensuring the durability of treated materials. Wang et al. [14] investigated the effects of silicate, phosphate, and mixed electrolytes on PEO coatings on aluminium, analysing surface and interface characteristics through FESEM, EDS, XRD, and XPS. Their findings revealed that silicate electrolytes formed Si-rich nodules, phosphate electrolytes produced Al_2_O_3_-rich protrusions, and mixed electrolytes combined matrix oxidation with deposition. Concurrently, Jiansheng et al. [15] used three-dimensional nonlinear finite element modelling to optimise heat treatment processes, simulating temperature, phase transformation, and stress–strain conditions to enhance quenching and carburising operations. Their simulations improved reliability, minimised distortion, and reduced processing times in nitriding and carburising, supporting efficient, robust production lines through CAD and CAE integration. Moreno et al. [16] developed a mathematical model for heating and cooling a steel workpiece, targeting a hardened surface with a ductile core to reduce fatigue. The model includes heating–cooling stages and viscoplasticity, enabling effective simulations for optimised treatment.

Despite the extensive research conducted on this topic by many researchers, gaps still need to be addressed. Key gaps in using PEO to enhance hardness in metallic alloys include limited control over plasma discharge mechanisms, coating uniformity, and layer brittleness. Accordingly, this experimental and mathematical modelling investigation was carried out to explore the effect of the electrolyte-plasma process on the diffusion saturation of LCS used in oil and gas fittings. This method notably reduces the saturation time to just a few minutes and allows for seamless integration with quenching in the same electrolyte, eliminating the need for reheating. The primary objective of this study is to assess the impact of PEO on low-alloy steel, aiming to improve productivity, processing efficiency, surface quality, and the overall structure and properties of the treated steel surface. This study’s novelty lies in determining the optimal parameters to achieve deep hardness with a defined depth, while also correlating experimental results with mathematical modelling to provide a more precise understanding of the process.

## 2. Materials and Experimental Work

**Figure 1:**
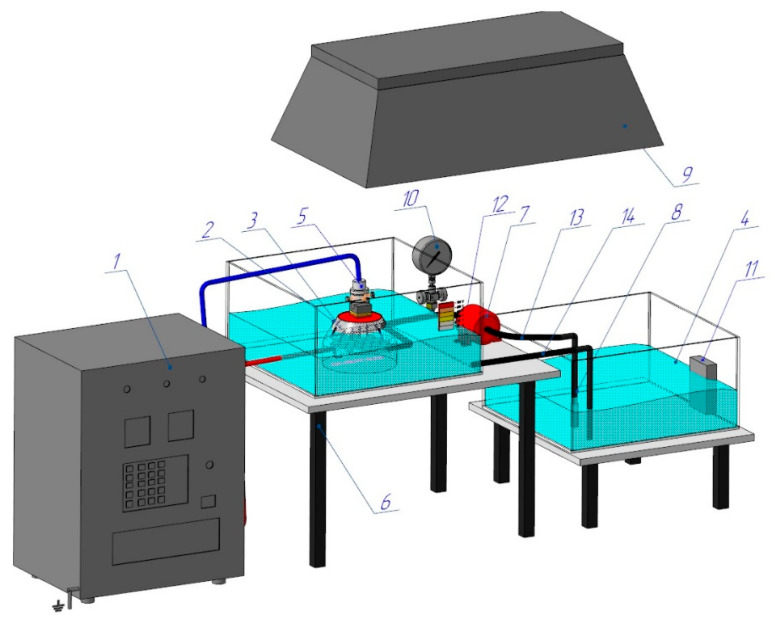
Experimental setup for plasma electrolyte oxidation. 1—power supply; 2—working bath; 3—conical nozzle; 4—reservoir; 5—clamping mechanism; 6—table; 7—pump; 8—filter; 9—hood; 10—manometer; 11—thermometer; 12—ball valve; 13, 14—high-pressure hoses.

Figure 1 shows a diagram of the PEO process, specifically designed and set up for this experiment. A programmable power supply (1) (hereinafter referred to as PS) converts the energy of a three-phase AC network with a frequency of 50 Hz into the energy of a pulsed single-phase high-voltage direct current.

**Figure 2:**
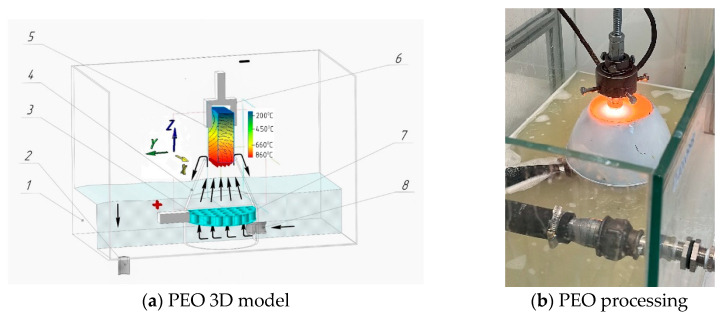
PEO process of a sample in a working bath. 1—working bath; 2—tube for return of electrolyte; 3—anode plate; 4—conical nozzle; 5—cathode—sample/detail; 6—clamping mechanism; 7—holes on the anode; 8—nozzle for electrolyte supply.

The workpiece is installed and securely clamped (5), which allows for the precise adjustment of the cathode’s (workpiece) depth of immersion into the Na_2_CO_3_ electrolyte solution. A conical nozzle (3) is the working device, generating vapour–air plasma between the liquid anode and the cathode (the workpiece). The electrolyte from the reservoir (4) is pumped by a high-pressure hose (14) into the conical nozzle (3), which houses the stainless steel anode made from 12Ch18N10T, equivalent to AISI 321. The electrolyte pressure is monitored by a pressure gauge (10) and adjusted using a ball valve (12), while the electrolyte’s temperature, maintained between 20 and 60 °C, is controlled by a thermometer (11). The used electrolyte is then cycled back into the reservoir (4) through a high-pressure hose (13). The parameters for the electrolyte-plasma treatment are determined experimentally: the voltage (U) is set to 200 V, the current (I) to 10 A, the processing time to 4–6 s, the hardening time to 4–10 s, and the total treatment time is 4 min. Quenching is performed directly in the electrolyte stream. Hardening of the workpiece occurs through periodic heating caused by the electric potential in the plasma layer, followed by surface cooling of the hardened workpiece, generated between the liquid electrode (electrolyte) and the cathode surface (workpiece) [1]. A critical component of the PEO process is the nozzle (3), as its design dictates the plasma density, the stability of ionisation, and the electrolyte’s velocity and flow characteristics. The conical nozzle, as shown in Figure 2a, is placed in a dielectric working bath 1. The anode (3) is designed as a cylindrical plate with transverse holes measuring 2 mm in diameter 7 [17]. The anode is inserted into the conical nozzle (4), while the hardened workpiece is the cathode (5). A 10% soda ash (Na_2_CO_3_ solution) electrolyte is fed from the reservoir through nozzle 8 into nozzle 4. During the process, the electrolyte circulates back to the reservoir via tube 2. When the power source is connected, a vapour–gas layer forms between the cathode and the liquid electrolyte, causing the film to boil [18]. During this brief period, the constituent components of the electrolyte become ionised, and the electrolyte-plasma is excited, as shown in Figure 2b.

Since plasma temperatures significantly exceed those required for structural phase transformations, finding the optimal modes of electrolyte-plasma treatment through experimental methods is essential. Quenching is performed in the electrolyte flow, with the best results achieved through cyclic processing [19]. The tests were conducted on specimens made from steel 20Ch (GOST 33260-2015) [2], with the following composition: C 0.17–0.23%, Si 0.17–0.37%, Mn 0.5–0.8%, and Cr 0.7–1.0%. The specimens were cut from a CES die to the dimensions of 10 × 10 × 25 mm. For comparison, after electrolytic-plasma hardening, a cross-section was prepared to investigate the hardness at deeper locations beneath the surface. A 1 mm thick diamond disc, immersed in a coolant, was used for cutting at low speeds (n = 350 rpm) and under a low load (m = 250 g). These conditions ensured that the sample did not experience significant deformation or thermal effects.

**Figure 3:**
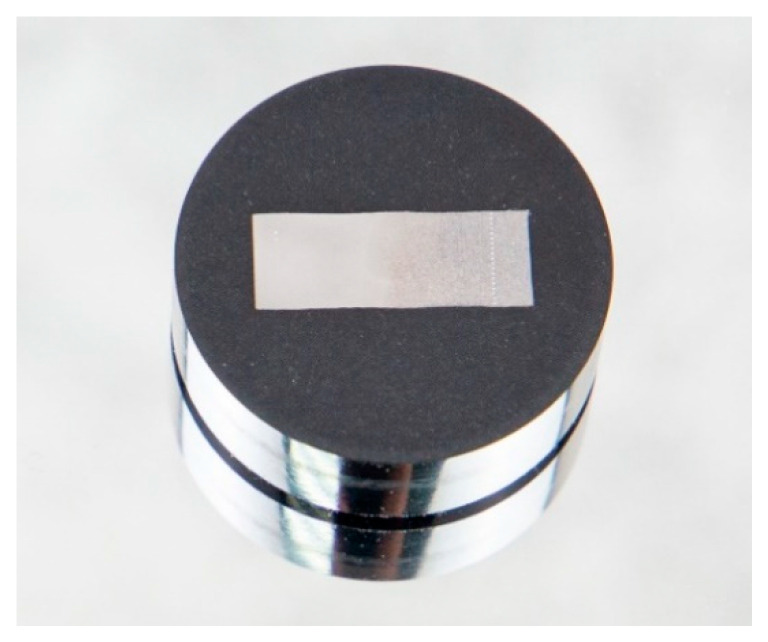
Sample preparation for the microstructure and cross-section analysis.

Figure 3 shows the samples used in metallographic and electron microscopic analysis. The samples were first ground, polished, and etched as received and after processes. After washing and drying, they were embedded in epoxy resin within a plastic mould and then subjected to further grinding and polishing until a mirror finish was obtained. The polished surfaces were then etched with a 5% nitric acid solution in ethyl alcohol, Nital, for 5–7 s. The degree of etching was monitored under a microscope, followed by fixation in ethyl alcohol and rinsing with running water. Metallographic substrate analysis was performed using an Axioscop-2MAT inverted reflected light photomicroscope (Dongguan, China), equipped with a Sony digital camera [20]. Microhardness was measured according to the Vickers method, following ST SEV 469-77 and ISO 6508-86 standards [21]. A conical diamond tip with an apex angle of 120° was used as the indenter, with hardness values rounded to the nearest 0.5 units [22]. The study of the topography and microstructure of the sample surfaces, as well as qualitative and quantitative elemental microanalysis, were conducted using a JSM-6390LV scanning electron microscope (SEM) from JEOL Ltd. (Tokyo, Japan), equipped with an INCA Energy Penta FET X3 energy-dispersive microanalysis system from OXFORD Instruments Analytical Limited (Oxford, UK).

**Figure 4:**
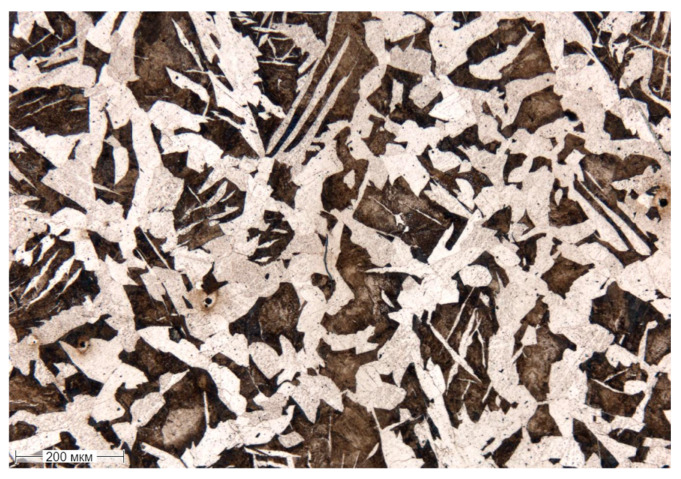
Microstructure of steel 20Ch in its initial state; optical microscopy.

Figure 4 shows the as-received hot-rolled microstructure of 20Ch steel, which consists primarily of ferrite and pearlite. The higher ferrite content is due to the low-carbon composition, with ferrite forming a soft, ductile matrix and pearlite providing strength through its lamellar structure of ferrite and cementite. As a result of the hot-rolling process, the grains are slightly elongated and appear coarse. Small chromium carbides are also observed.

### 2.1. PEO Process Simulation

PEO involves immersing the metal part, which serves as the anode, in an electrolyte solution containing compounds like sodium carbonate (Na_2_CO_3_) or sodium hydroxide (NaOH). A high-voltage power supply is applied, leading to the formation of plasma discharges at the metal–electrolyte interface once the voltage exceeds a critical threshold. These plasma microdischarges generate localised extreme temperatures (~6000 K), which facilitate rapid oxidation of the metal surface. The oxide layer grows both inwardly, into the metal substrate, and outwardly, into the electrolyte, creating a multi-layer structure consisting of an outer porous layer and a dense inner layer. The outer layer allows further ion exchange from the electrolyte, while the dense layer provides excellent mechanical strength, wear resistance, and corrosion protection. The process parameters, such as voltage, current density, and electrolyte composition, significantly influence the layer’s thickness, hardness, and uniformity. Once the desired layer properties are achieved, the power supply is turned off, and the part is allowed to cool, often in the electrolyte.

**Figure 5:**
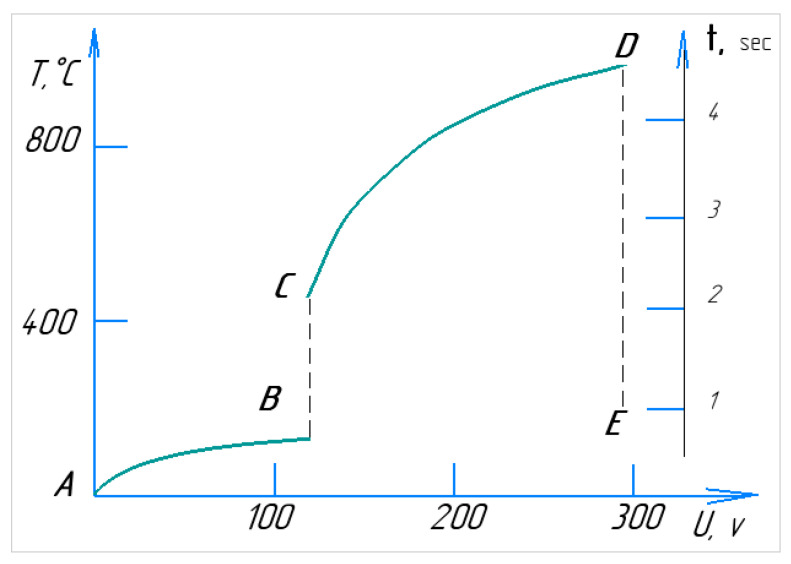
Volt–temperature characteristics of PEO, in the time interval of one cycle.

Figure 5 illustrates the volt–temperature characteristics of the PEO process, along with the real-time progression of a single cycle. When the electrolyte circulates from the reservoir to the nozzle and through the working bath at an initial temperature of around 20 °C, plasma is expected to form within a few seconds [8]. At low voltages (section AB), the cathode immersed in the electrolyte encounters electrical resistance. As the voltage increases, localised resistance around the sample heats the electrolyte in the contact zone, leading to boiling and the onset of current interruption (section BC). In this region, the current flow becomes pulsed due to periodic condensation and the formation of a vapour layer around the cathode/workpiece [23]. This stage is characterised by spark discharges, often accompanied by crackling and luminescence. At point C, the electrolyte-plasma stabilises, and the system shifts into a heating phase (section CD), in which the temperature rises. As the voltage increases to 150–280 V, the power dissipated in the plasma increases, causing a further temperature rise [24]. Stage DE represents the point at which the power supply is turned off, causing the voltage to drop to 0, while the temperature decreases over time.

The section DE is marked by a sharp drop in the cathode temperature, caused by the cooling effect of the circulating electrolyte. The most critical parameters of the PEO process are temperature and heating rate. Due to its high heating rate, PEO is classified as a rapid chemical–thermal treatment method [25]. For the cathodic version of PEO, at a constant voltage, the heating rate of the part can reach up to 200 °C per second [26], while for the anodic version, it can go up to 250 °C per second [27]. The heating temperature primarily depends on factors such as applied voltage, electrode size, and the temperature and flow rate of the electrolyte. In the heating mode, the anode temperature peaks relative to the applied voltage. Increasing the diameter of the anode decreases its temperature at a constant voltage while increasing its height (or immersion depth) results in a higher temperature. This is due to the unique distribution of heat flows within the system. As a result, the maximum heating temperature of the sample in the Na_2_CO_3_ solution was achieved at the lowest electrolyte temperature, while the minimum heating temperature occurred at the highest.

Table 1 presents the factor ranges of the numerical values (*k*i) for the parameters under study, which are key to optimising the heating and heat treatment process during electrolytic-plasma treatment. This optimisation can be represented as a function of Q (*K*_1_, *K*_2_, *K*_3_, *K*_4_).

### 2.2. Mathematical Modelling

To perform a comparative analysis of the influence of technological parameters on material strengthening, a decision was made to adopt a three-factor experimental model, fixing the number of processing cycles at *k*4 = 30 cycles. Through experimentation, the primary factors affecting the quality of steel hardening during PEO were identified as heating time, hardening time, and applied voltage. The heating and heat treatment processes during PEO are directly linked to the variation in the material’s temperature (T), which follows the thermal conductivity Equation (1) [28].

(1) $$\frac{dT}{dt}=\lambda ∆T$$

where *λ* is the coefficient of thermal conductivity, and *t* is time.

(2) $$∆T=\frac{d^{2}T}{dx^{2}}+\frac{d^{2}T}{dy^{2}}+\frac{d^{2}T}{dz^{2}}$$

where the *x*, *y*, *z* coordinates allow the sample’s spatial case to be considered, as shown in Figure 2a.

For objects of different shapes, different coordinate systems can be considered, and Equation (1) can be represented as

(3) $$\frac{dT}{dt}=\frac{1}{x^{n}}\frac{d}{dx}\left(x^{n}\lambda \frac{dT}{dx}\right),$$

where for *n* = 0, Equation (3) is investigated in a rectangular coordinate system; for *n* = 1, in a cylindrical coordination system; and for *n* = 2, in a spherical coordinate system.

The uniqueness of the solution is provided by the initial and boundary conditions.

The boundary conditions in the general setting are as follows:

(4) $${\left.\left(A_{1}T+A_{2}\frac{dT}{dx}\right)\right|}_{x=0}=A;\;{\left.\left(B_{1}T+B_{2}\frac{dT}{dx}\right)\right|}_{x=0}=B;\;{\left.\left(C_{1}T+C_{2}\frac{dT}{dx}\right)\right|}_{x=1}=C;\;{\left.\left(D_{1}T+D_{2}\frac{dT}{dx}\right)\right|}_{x=0}=D.$$

By setting parameters *A*_1_, *A*_2_, *B*_1_, *B*_2_, *C*_1_, *C_2_*, *D*_1_, *D*_2_ in Equation (4), the boundary conditions of the problem can be varied. Table 2 summarises the influence of the PEO modes and the quality indicators of steel 20Ch hardening, which depend on parameters such as the heating time T_heating_ from ionised plasma, the quenching time T_quenching_, and the DC voltage U between the electrodes.

The heating temperature is the main parameter of phase transformations, for steel 20Ch, which is 840–860 °C [29]. When the ionised plasma is excited (the plasma temperature is in the range of 6000 K to 30,000 K), a vapour–gas layer appears on the sample’s surface, resulting in the dissociation of the electrolyte.

**Figure 6:**
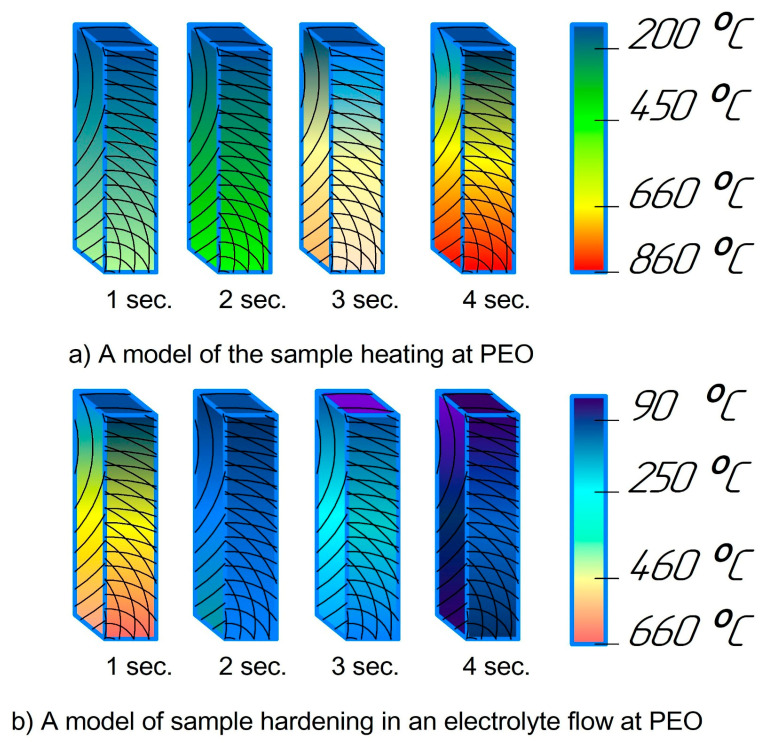
A mathematical model estimation of the temperature profile dependence on the quenching time of the modelled steels in the electrolyte.

Figure 6a shows a simulation, using KOMPAS-3D version 20.0.0.3002, in which increasing plasma temperature rapidly heats the sample within a few seconds. Figure 6b illustrates the quenching process when the power source is turned off, with the sample being cooled in the electrolyte flow.

Taking into account the accepted assumptions, the thermal conductivity equation can be reduced to the thermal equation of the two-phase zone model (5):

(5) $$\Psi \left(T\right)\frac{dT}{dt}=\lambda \left(\varepsilon_{V,}\varepsilon_{\sigma }\right)\nabla^{2}T+F(qL)$$

where Ψ is the dimensionless effective heat capacity, which takes into account the release of the latent heat of the phase transition; *T* is the absolute temperature; *t* is time; λ is the coefficient of thermal conductivity; *ε_V_* and *ε_σ_* are the density characteristics of the hardened layer, defined as the volume fraction of the density and the fraction of the hardened layer in a flat section, respectively; and *F* = α*qL* is the intensity of the volumetric heat source associated with the power of the plasma effect at different depths y of the powder layer. Here, *qL* and α are the plasma radiation energy flux density and the absorption coefficient of light radiation in the local volume of the hardened layer, respectively. The coefficient α depends on both the temperature and the phase composition of the local volume, determining in the model the change in the penetration depth of plasma radiation during particle melting and changes in the morphology of the body being hardened.

The vapour–gas layer prevents electrolytes from reaching the overheated surface. This slows down the cooling rate, preventing the formation of thermal (hardening) cracks. As a result, the steel’s service life is increased.

A mathematical model was developed to describe the change in the key parameters of the PEO hardening process, namely the heating temperature *T*. The logarithmic dependence of temperature *T* on the main factors is expressed by the following regression equation, with time expressed as t:

(6) $$\ln(T)=A\cdot k1\cdot \ln(t_{heat})+B\cdot k2\cdot \ln(U)+C\cdot k3\cdot \ln(t_{cool})$$

Parameter D modes are excluded because the sample melts from the plasma temperature. Coefficients for Equation (6) were found using a logarithm in the Deductor Studio Academic program. Then the Equation (6) of the dependence of the heating temperature on the heating time, cooling time, and voltage was transformed into a power law (6):

(7) $$T=4.5\cdot t_{heat}^{2}+4.8\cdot U-25\cdot t_{cool}$$

where *T* is the temperature of steel heating, *t*_heat_ is the heating time, *t*_cool_ is the cooling time in the electrolyte flow, and *U* is the voltage [30].

The experimentally determined optimal modes of steel hardening by the PEO method (*t*_heat_ = 4 s, *t*_cool_ = 4 s, *U* = 200 V) correlate well with the established relationship (7).

Initially, a series of experiments were conducted to determine the optimal processing parameters, with the results showing the maximum and minimum values presented in Table 1. To derive the regression formula, the number of cycles was excluded as it was treated as a constant parameter. This exclusion was justified because solving a four-factor equation would be overly cumbersome, and practically, the number of cycles does not function as a variable factor since it remains constant throughout the experiments. This approach allowed us to concentrate on the factors that directly influence the processing parameters, without oversimplifying the analysis.

This mode shows the sample/part being heated to 840–860 °C, sufficient for the cathodic phase transformation of steel 20Ch.

## 3. Results and Discussion

Under traditional production conditions, saturating the surface of the steel alloy with carbon and achieving a similar microstructure requires a significantly longer time and a higher energy consumption. In contrast, the PEO process was completed in just 4 min of cyclic processing. Each cycle consisted of 4 s of plasma heating followed by 4 s of quenching in an electrolyte flow, resulting in 30 cycles. Previous studies [31] have experimentally determined the optimal modes for PEO processing and highlighted metal saturation with carbon from the decomposition of ions from the Na_2_CO_3_ electrolyte components and the anode. Diffusion saturation of steel with carbon and other elements can occur not only from the electrolyte, but also from the elements of the anode, which, in this case, was made of stainless steel 12Ch18N10T (GOST 2590-2006, X6CrNiTi18-10) [32].

**Figure 7:**
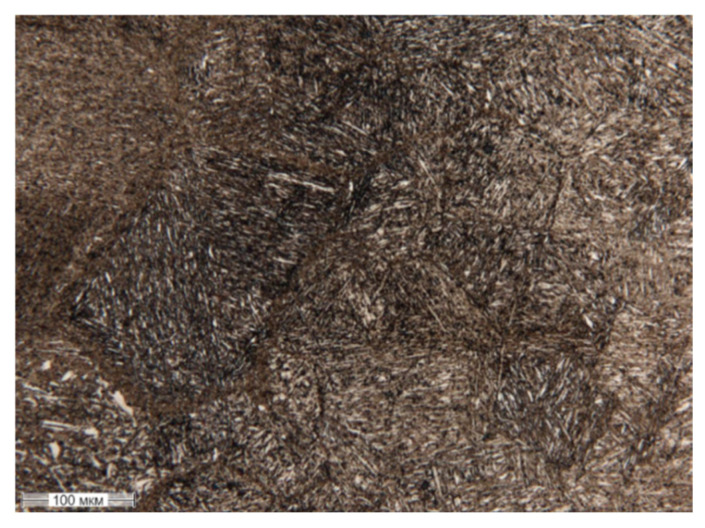
Microstructure of the surface layer of steel 20Ch after PEO, U = 200 V, t = 4 min.

Figure 7 shows the microstructure of a 20Ch steel sample treated with PEO, revealing the presence of acicular quenched martensite.

**Figure 8:**
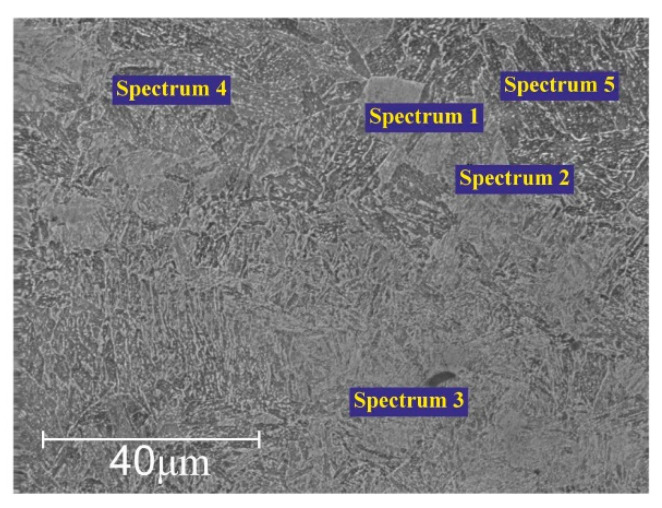
EDS analysis, using SEM, after PEO.

Figure 8 presents the Energy Dispersive X-ray Spectroscopy (EDS) analysis for elemental microanalysis at different points, highlighting the changes in the chemical composition of 20Ch steel after electrolytic-plasma hardening. Table 3 shows that during electrolytic-plasma hardening, the content of alloying elements like chromium and nickel in 20Ch steel increases, improving its wear resistance and mechanical properties. Sodium is alloyed into the steel through reactions with an electrolyte containing calcined soda (Na_2_CO_3_). The thermo-cyclic hardening process not only modifies the chemical composition but can also alter the microstructure of the steel, enhancing its strength characteristics. These changes can positively impact the steel’s performance properties, such as hardness and wear resistance.

On average, the carbon content of the as-received alloy was 0.17–0.23%. However, after the PEO process, an increase in carbon content was observed (see Table 3). This can be attributed to the penetration of carbon ions from soda ash (Na_2_CO_3_) and the anode into the stainless steel cathode.

**Figure 9:**
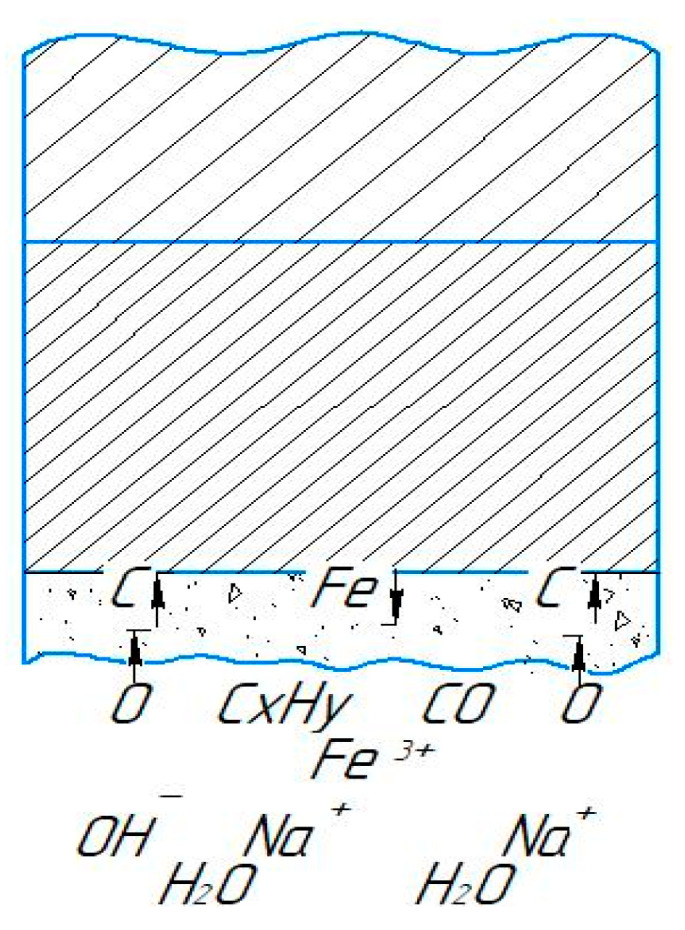
An illustration of the electrolytic-plasma treatment of the workpiece surface.

Figure 9 illustrates models that show the positive effect of the electric field on the cathode surface in facilitating the transfer of carbon ions. The figure illustrates several factors that govern the requirements for electrolyte composition used in PEO. The electrolyte must be able to heat the parts to temperatures high enough to enable effective carbon diffusion. Additionally, the solution must have a minimum electrical conductivity to generate the PEO effect. The saturating potential of different electrolytes depends on the decomposition reactions of carbon-containing components and the adsorption of the resulting decomposition products on the treated surface [33].

The electrolyte circulation rate plays a crucial role in controlling the intensity of stirring, cooling, and quenching of the sample [34]. The heat transfer dynamics in the near-cathode zone, influenced by the hydrodynamic conditions within the conical nozzle, significantly impact the release and transfer of saturating components to the surface of the workpiece. These dynamics are also influenced by the composition of the electrolytes, which, in combination with the processing parameters, determine the material properties achieved after PEO.

**Figure 10:**
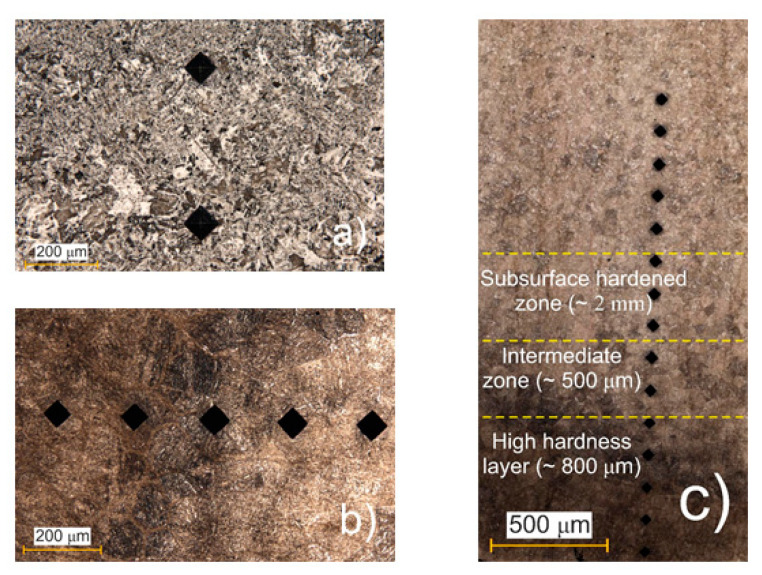
(**a**) The surface of the as-received alloy before PEO, (**b**) the surface of the alloy after PEO treatment, and (**c**) a cross-section of the alloy showing the high-hardness region (surface layer), medium-hardness region (intermediate zone), and low-hardness region (subsurface zone).

A highly concentrated plasma temperature forms on the steel substrate surface, creating a steep carbon concentration gradient. The PEO experiments were conducted on 20Ch alloy, and the cross-section was analysed after the process, which involved 30 hardening cycles. Figure 10a,b show the surface with microhardness indentations of the as-received alloy before and after PEO, respectively. Figure 10c shows the cross-section of the alloy after PEO processing. From the surface to a depth of approximately 3 mm, distinct structural and colour changes were observed. The outermost layer, referred to as the High-Hardness Layer (~800 µm), appeared dark and exhibited maximum hardness due to the plasma temperature (around 6000 K) and rapid cooling in the electrolyte. Beneath this, the Intermediate Zone (~500 µm) displayed a mixed dark- and light-coloured appearance, representing a transition region with moderate structural modification. Beyond this, the Subsurface Hardened Zone (~2 mm) was characterised by a uniformly lighter colour and exhibited higher hardness than the as-received microstructure. This increase in hardness was due to the formation of martensite through quenching, though it was less pronounced than in the surface layer.

The cyclic quenching in the electrolyte flow generates a thermal effect on the steel’s microstructure, leading to phase transformation and the formation of fine-acicular martensite. Although no sharply defined boundaries are present between sections, the total depth of the hardened layer reaches up to 3 mm, gradually transitioning into the original pearlite–ferrite structure [16]. Given the small size and thinness of the sample, varying the applied voltage allows for precise control of the cathode’s (workpiece) temperature, ranging from 400 to 1100 °C [15]. This temperature exhibits an almost linear dependence on the applied voltage [35]. The high rates of electrolytic-plasma heating reduce the time required to reach the target temperature and speed up the formation of diffusion layers, positively influencing certain stages of the process. While slow heating promotes grain growth, rapid heating can increase the temperature more quickly, thereby accelerating diffusion.

Cathodic cyclic hardening of structural steels using PEO in an electrolyte containing 10% soda ash at 840–860 °C can double the hardness of the material in just 4 min of treatment, significantly faster than traditional carburising in a solid carburiser followed by quenching. Additionally, this method demonstrates a potential reduction in heat treatment time, which in turn lowers energy consumption for heating industrial furnaces and ultimately reduces the overall cost of part manufacturing.

**Figure 11:**
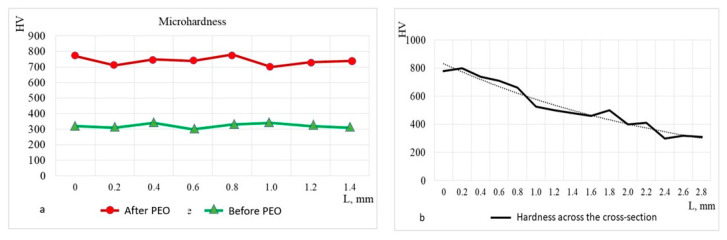
Variations in microhardness were measured on 20Ch steel: (**a**) Vickers hardness values for the surfaces before and after PEO, and (**b**) microhardness values across the cross-sectional depths after PEO.

Figure 11 presents the results of microhardness measurements taken from the surface and cross-section of the sample at 200 μm intervals. Figure 11a shows the surface hardness over a length of approximately 1.2 mm, comparing the values before and after PEO. The results indicate an increase in hardness from an initial value of an average of HV 300 before PEO, to an average of HV 750 after PEO. Figure 11b illustrates the reduction in hardness from the surface (~HV 750), across the cross-sectional depth. At a depth of 2.8 mm, the hardness decreases to match the surface hardness of the as-received alloy. The hardness distribution after PEO treatment closely aligns with the carbon concentration distribution in steel 20Ch, characteristic of hypo-eutectoid steel, and corresponds to the saturating capability of the electrolytes used [36]. This increase is attributed to the maximum total concentration of carbon, which forms a supersaturated solution in austenite at the saturation temperature and transforms into martensite after quenching. To prevent potential calcination and increased brittleness, it is recommended that PEO parameters, including cyclic quenching by electrolyte flow, are determined empirically and confirmed through calculations as part of experiment planning.

Introducing relatively new PEO technology offers the opportunity to gain new insights into electrolyte-plasma hardening principles. From a practical perspective, understanding the PEO mechanism is essential for making informed decisions on processing modes and electrolyte compositions, and for designing the necessary equipment.

## 4. Conclusions

(a) Effectiveness of PEO for Surface Hardening: Our experimental investigation of PEO for LCS alloy type 20Ch, combined with a mathematical model using thermal conductivity equations and regression analysis, demonstrates significant potential for PEO as a surface hardening process for steels.
(b) Reduced Processing Time: The PEO treatment in our study showed that the workpiece’s heating time from the high-temperature electrolyte-plasma was only 4 min—substantially shorter than the time required for traditional carburising followed by quenching.
(c) Formation of a Carbon-Saturated Layer: PEO treatment formed a carbon-saturated, martensitic hardened layer on the sample’s surface. This layer smoothly transitioned into the original pearlite–ferrite structure.
(d) During the electrolytic-plasma hardening process, an interaction occured between the 12Ch18N10T steel anode and the electrolyte, leading to the diffusion of chromium, nickel, and sodium elements onto the surface of the cathode sample.
(e) Improved Surface Hardness: The surface microhardness of the 20Ch steel sample after PEO increased by a factor of two compared to its initial state, indicating a significant enhancement of its material properties.

PEO has been validated as a cost-effective and energy-efficient surface hardening process with reduced processing time. This positions it as a competitive alternative to traditional heat treatment methods, which often require modernisation to enhance efficiency and maintain competitiveness.

## Figures and Tables

**Table 1:** Variable factors during plasma electrolyte oxidation.

Factors	Physical Values of Factors	Factor Levels	
		Min	Max
*K*_1_	The heating time of the workpiece from the plasma temperature, s	1	15
*K*_2_	DC voltage, V	180	300
*K*_3_	Cooling time in electrolyte flow, s	1	10
*K*_4_	Number of processing cycles	20	40

**Table 2:** PEO modes during hardening of steel 20Ch.

Parameters	PEO Processing Modes			
	A	B	C	D
T_heating_, s	2	4	8	15
T_hardening_, s	2	4	8	15
U, V	180	200	250	300

**Table 3:** Elemental chemical composition, in wt%, of 20Ch steel after PEO.

Spectrum	C%	Cr%	Ni%	Mn%	Si%	P%	S%
1	0.23	1.19	0.21	0.68	0.25	0.019	0.034
2	0.29	1.09	0.28	0.65	0.32	0.025	0.027
3	0.28	1.53	0.21	0.76	0.36	0.031	0.029
4	0.35	1.23	0.13	0.80	0.28	0.018	0.022
5	0.31	1.05	0.29	0.91	0.31	0.029	0.031

## Data Availability

The original contributions presented in this study are included in the article. Further inquiries can be directed to the corresponding author.
